# Population pharmacokinetics of cefotaxime in intensive care patients

**DOI:** 10.1007/s00228-021-03218-6

**Published:** 2021-10-01

**Authors:** Maria Swartling, Anna-Karin Smekal, Mia Furebring, Miklos Lipcsey, Siv Jönsson, Elisabet I. Nielsen

**Affiliations:** 1 Department of Pharmacy, Uppsala University, Uppsala, Sweden; 2 Department of Surgical Sciences, Anaesthesiology and Intensive Care, Uppsala University, Uppsala, Sweden; 3 Department of Medical Sciences, Infectious Medicine, Uppsala University, Uppsala, Sweden

**Keywords:** Population pharmacokinetics, Modelling, Cefotaxime, Critically ill

## Abstract

**Purpose:**

To characterise the pharmacokinetics and associated variability of cefotaxime in adult intensive care unit (ICU) patients and to assess the impact of patient covariates.

**Methods:**

This work was based on data from cefotaxime-treated patients included in the ACCIS (Antibiotic Concentrations in Critical Ill ICU Patients in Sweden) study. Clinical data from 51 patients at seven different ICUs in Sweden, given cefotaxime (1000–3000 mg given 2–6 times daily), were collected from the first day of treatment for up to three consecutive days. In total, 263 cefotaxime samples were included in the population pharmacokinetic analysis.

**Results:**

A two-compartment model with linear elimination, proportional residual error and inter-individual variability (IIV) on clearance and central volume of distribution best described the data. The typical individual was 64 years, with body weight at ICU admission of 92 kg and estimated creatinine clearance of 94 mL/min. The resulting typical value of clearance was 11.1 L/h, central volume of distribution 5.1 L, peripheral volume of distribution 18.2 L and inter-compartmental clearance 14.5 L/h. The estimated creatinine clearance proved to be a significant covariate on clearance (*p* < 0.001), reducing IIV from 68 to 49%.

**Conclusion:**

A population pharmacokinetic model was developed to describe cefotaxime pharmacokinetics and associated variability in adult ICU patients. The estimated creatinine clearance partly explained the IIV in cefotaxime clearance. However, the remaining unexplained IIV is high and suggests a need for dose individualisation using therapeutic drug monitoring where the developed model, after evaluation of predictive performance, may provide support.

**Supplementary Information:**

The online version contains supplementary material available at 10.1007/s00228-021-03218-6.

## Introduction

Infections are common in critically ill patients and associated with considerable morbidity, mortality and healthcare costs [1]. Cefotaxime is a broad-spectrum beta-lactam antibiotic used in the empirical treatment of several serious infections, and it is currently the most commonly used intravenous cephalosporin in Sweden according to national statistics on antibiotic use.

Cefotaxime disposition has previously been described [2] by a two-compartment model, with linear pharmacokinetics (PK). The half-life in healthy volunteers is reported to be around 1 h, with 80% of an intravenous dose excreted in urine whereas 20% is recovered in faeces following biliary excretion. Cefotaxime is partially metabolised to desacetyl-cefotaxime, a metabolite with antibacterial activity reported to be two to 16 times less than that of cefotaxime depending on bacterial species [3].

Critically ill patients with sepsis frequently have significant physiological alterations that can lead to changed PK of the hydrophilic beta-lactams [4]. A large increase in volume of distribution (*V*) has been observed, and this effect is especially pronounced in burn patients [5]. Further, critically ill patients are at risk for acute kidney injury and reduced clearance (*CL*) of renally excreted antibiotics [4] as well as augmented renal clearance (ARC) [6].

The high inter-individual variability (IIV) of PK parameters in critically ill patients indicates the need for a more individualised approach to dosing. Population PK models describe the typical PK in a population as well as variability between individuals and may include covariates that help explain the variability [7]. Such models can be used before the start of treatment to predict a dosing regimen that maximises the chances of reaching PK-related treatment targets. They can also be used to obtain individual PK parameters based on measured drug concentrations, and subsequently for individual predictions and dose selection. In the case of cefotaxime, there are published population PK models for elderly patients [8] and paediatric patients [9–12], but to our knowledge, no such model for adult intensive care unit (ICU) patients has been published.

The aim of this study was to characterise the PK and associated variability of cefotaxime in adult ICU patients, and to assess the impact of patient covariates relevant for clinical practice, using a nonlinear mixed-effects modelling approach.

## Materials and methods

### Study design

The current analysis is a sub-study to ACCIS (Antibiotic Concentrations in Critical Ill ICU Patients in Sweden), a prospective observational multi-centre PK study conducted at seven ICUs, including one burn unit, in five Swedish hospitals between December 2015 and July 2017 (Trial ID ACTRN12616000167460). The Uppsala regional ethical review board approved the study (EPN Dnr 2015/135), and informed consent was obtained from the patient. The next of kin was consulted if the patient was unable to give consent. The study was performed in line with the principles of the Declaration of Helsinki and its revisions.

### Patient population

Patients above the age of 18 years admitted to the ICUs and treated with cefotaxime for a proven or suspected infection were included in the study. Patients were included within 24 h from initiation of cefotaxime treatment. Pregnant patients and patients with treatment restrictions were excluded in the study. Additionally, patients on renal replacement therapy were excluded in this current analysis. Demographic and covariate data were obtained from the patient medical records.

### Dosing regimen and sampling

The cefotaxime dose was chosen on discretion of the treating physician, where the recommended dose ranges from 1000 to 2000 mg three times daily (t.i.d.) depending on the severity of the infection and 3000 mg four times daily for meningitis. An additional dose in the middle of the first dose interval is recommended in sepsis according to the national guidelines by the Swedish Society of Infectious Diseases. Cefotaxime was administered intravenously as a short infusion over 5 min. Blood samples for measurement of cefotaxime concentrations were collected twice during one dosing interval per day, at mid-point and just before next dose, from the first day of treatment for up to three consecutive days if cefotaxime treatment was maintained. Nurses registered the exact time points for blood sample collection and dose administration in a case report form.

### Cefotaxime analysis

Samples were centrifuged for 7 min at 2400 g within 3 h of collection, and serum was stored at − 70 °C for later analysis. Total serum cefotaxime concentrations were determined by LC–MS/MS at the Department of Clinical Pharmacology, Karolinska University Hospital Huddinge, Stockholm, Sweden. The bioanalytical method was validated according to the European Medicines Agency Guideline on bioanalytical method validation and has been previously described for beta-lactams [13]. The range of quantification was 0.50–50 mg/L with a total coefficient of variation of 11.6% at the lower limit of quantification (LLOQ) and ≤ 6.0% in the quantification range. Accuracy was within − 6.8 to + 4.4%.

### Pharmacokinetic modelling

A population PK model was developed using NONMEM (version 7.4, Icon Development Solutions, Hanover, MD, USA) [14] and Pearl-Speaks-NONMEM [15] to assist with the execution of models. The first-order conditional estimation method with interaction was used for parameter estimation. R version 3.5 (R Foundation for Statistical Computing, Vienna, Austria) was used for data management and the xpose4 package [15] for model diagnostics and evaluation.

For hierarchical models, the likelihood ratio test was used to evaluate statistical significance for the inclusion of additional parameters in the model, with the assumption that the difference in objective function value (OFV) between two hierarchical models is *χ*^2^-distributed. A reduction of OFV of 3.84 between two models with one parameter difference was considered statistically significant with a *p*-value of 0.05, whereas a reduction of OFV of 10.83 was considered significant with a *p*-value of 0.001. Model selection was further guided by basic goodness-of-fit plots and simulation-based prediction-corrected visual predictive checks (pcVPCs) [16]. A nonparametric bootstrap (*n* = 2000) was used to evaluate the stability and the confidence intervals (CIs) for the final parameter estimates. Further, maximum a posteriori (MAP) estimation was performed using the final model with observations in the dataset to evaluate the precision of the individual parameter estimates.

One- and two-compartment models with linear elimination were evaluated. To describe the variability between patients, IIV terms assuming log-normally distributed individual parameters were tested and included if statistically significant. Different residual error models were evaluated (additive, proportional and slope-intercept).

Only patient covariates that are readily available in the clinical setting were considered for testing to explain part of the random parameter variability. As body size is expected to influence PK parameters [17] and as increased weight due to treatment with resuscitation fluids might reflect *V* in a critically ill patient, weight at ICU admission was added before other covariates were included as a time-independent mechanistic covariate in line with allometric principles [17]. As cefotaxime is renally cleared to a large part, estimated creatinine clearance (*eCLcr*), derived from the Cockcroft-Gault formula [18], was tested as a covariate on *CL* as a time-dependent continuous covariate. As the PK parameters might change during the course of treatment, day of antibiotic treatment was tested as a time-dependent categorical covariate on volume parameters and *CL*. Further, as *V* in burn patients might be different from that in other ICU patients [5], the burn unit patient subgroup was tested as a time-independent categorical covariate on volume parameters. Finally, Simplified Acute Physiology Score 3 (SAPS3) was tested as a continuous covariate in an attempt to evaluate non-renal factors on *CL*. A stepwise forward inclusion procedure was used (*p* < 0.05) to build the full model, and stepwise backward elimination (*p* < 0.001) to determine the final model.

Time-dependent continuous covariates were carried forward within individuals to the next observation, without interpolation between measurements. In case of missing data in the beginning of the time series, the first observation was carried backwards. Missing data for the time-independent covariate body weight at ICU admission (7 individuals) were imputed using linear regression with weight before ICU admission. Missing data for SAPS3 (2 individuals) were imputed using the population mean. Fifteen cefotaxime concentrations (6% of the total number, with no consecutive concentrations below LLOQ) from eight individuals were below LLOQ and were set to LLOQ/2 [19].

Continuous covariates were incorporated and evaluated in the model using different relationships (linear, piecewise linear, exponential or power). Linear and piecewise linear relationships were parameterised as Typical *CL* = *θ*_median_ × [1 + *θ*_cov_ × (*X*_*i*_ − *X*_median_)], where *θ*_median_ is the typical *CL* at the covariate median and *θ*_cov_ is the covariate effect, in this case as a fractional change per unit different from the population covariate median (*X*_median_); *X*_*i*_ is the covariate value for the individual. For the piecewise linear model, different slope parameters in different parts of the covariate range with a common breakpoint was estimated and different breakpoints were evaluated in a stepwise manner. Categorical covariates were incorporated as an estimated fractional change [1 + *θ*_cov_] in typical value from the reference category.

## Results

### Patient characteristics and data

**Fig. 1:**
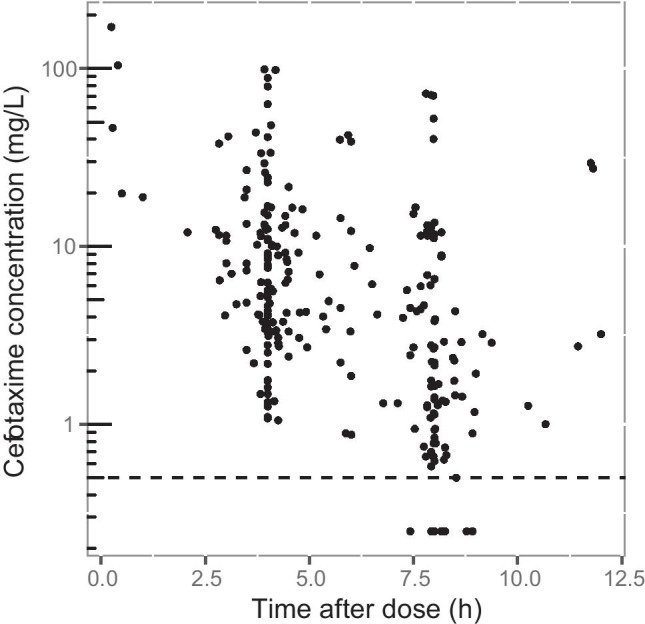
A scatter plot of observed serum cefotaxime concentrations from 51 patients versus time after dose. Dots are the individual concentrations; dashed line marks the lower limit of quantification (0.5 mg/L)

Fifty-one ICU patients, 18 treated in a burn unit ICU and 33 in general ICU units, were included in the current study with a total of 263 cefotaxime samples and a median of 6 (range 2–6) samples per patient. Four samples were excluded from the original dataset due to reported erroneous high concentrations at a supposed trough time point. Table 1 shows a summary of the patient characteristics and collected data. Figure 1 illustrates the observed serum cefotaxime concentrations in relation to time after dose. The most common causes of infection were lower respiratory tract infections in 59% (30/51) of the patients followed by skin and soft tissue infections in 25% (13/51) and urinary tract infections in 8% (4/51). The administered cefotaxime doses ranged from 1000 to 3000 mg given 2–6 times daily, with the most common regimen being 1000 mg t.i.d, followed by 2000 mg t.i.d. An extra dose in the first dose interval was given in four cases.

**Table 1:** Overview of patient characteristics and observed data for included patients (*n* = 51)

**Characteristic**	**Values** Median (IQR) [range] or number (%)
**Time-independent variables**	
Age (years)	64 (50–73) [23–90]
Female	18 (35%)
SAPS3 at admission to ICU	55 (47–63) [25–89]
Body weight before ICU admission (kg)	87 (79–99) [55–123]
Body weight at ICU admission (kg)	92 (81–102) [55–124]
**Time-dependent variables**	
*eCLcr* (mL/min), treatment days 1–3	94 (65–138) [5–258]
Treatment day 1	85 (52–133) [5–208]
Treatment day 2	97 (65–130) [16–207]
Treatment day 3	105 (80–159) [19–258]
Serum cefotaxime trough^a^ concentration (mg/L), treatment days 1–3 (*n* = 112)	1.8 (0.8–5.7) [0.25–72]
Treatment day 1 (*n* = 42)	2.6 (0.9–7.5) [0.25–72]
Treatment day 2 (*n* = 43)	2.2 (0.8–4.4) [0.25–70]
Treatment day 3 (*n* = 27)	1.4 (0.7–2.8) [0.25–12.2]

*IQR*, interquartile range; *SAPS3*, Simplified Acute Physiology Score 3; *ICU*, intensive care unit; *eCLcr*, estimated creatinine clearance, determined using the Cockcroft and Gault formula and body weight at ICU admission

^a^Trough defined as a sample drawn ≤ 1 h before next scheduled dose

### Pharmacokinetic modelling

**Fig. 2:**
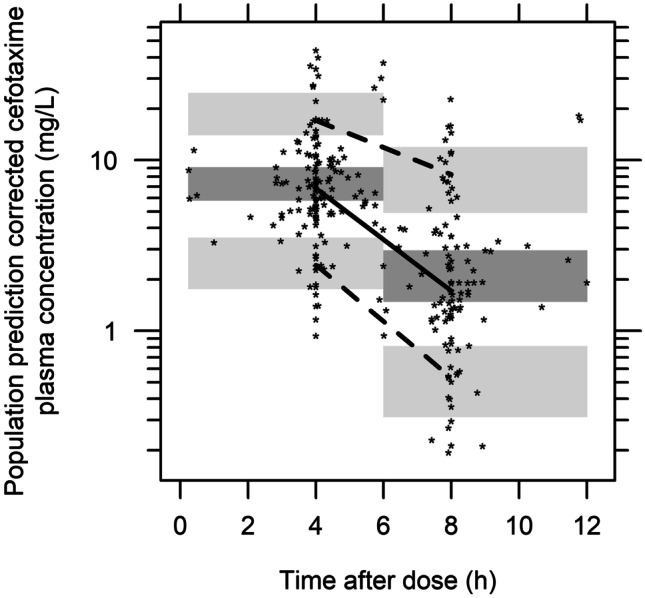
Population prediction-corrected visual predictive check (pcVPC) for serum cefotaxime concentrations versus time after dose. Dots are the individual concentrations; solid line is the median of the observed data; dashed lines are the 10th and 90th percentiles of the observed data; shaded areas are the 95% confidence intervals around the median (dark grey) and the 10th and 90th prediction intervals (light grey) of the simulated data

A two-compartment model with linear elimination from the central compartment and a proportional residual error model best described the data﻿. IIV was possible to estimate for *CL* and central volume of distribution (*Vc*), and was found to be highly positively correlated. Therefore, the same IIV term was used for *Vc* and *CL*, with a scaling factor for *Vc* to allow for different magnitudes of IIV (Table 2, Fig. 2).

**Fig. 3:**
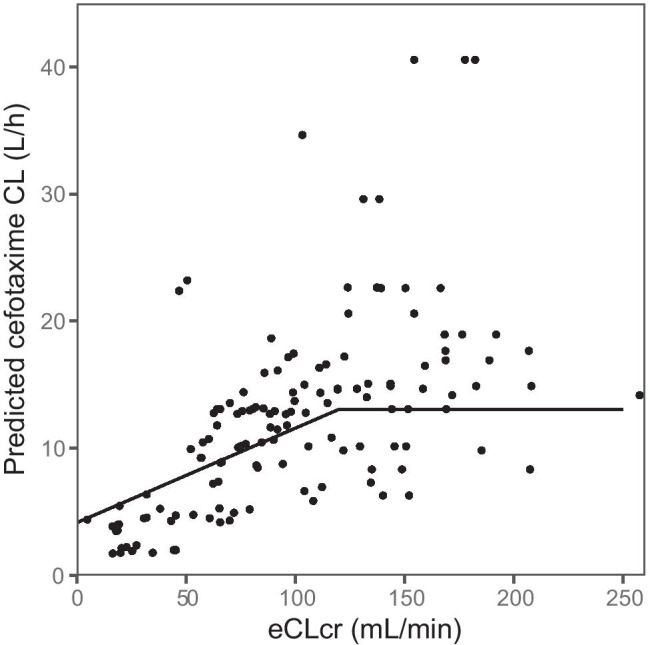
Illustration of the covariate effect of estimated creatinine clearance (*eCLcr*) on cefotaxime clearance (*CL*). Dots are individual *CL* data points; black lines mark the different linear change in the piecewise linear model with a common breakpoint at *eCLcr* 120 mL/min

The estimated *CLcr* was included as a piecewise linear covariate on *CL* with a fixed slope of zero at values above 120 mL/min, as the estimated slope approached zero. This equals truncating *eCLcr* at high values, a strategy applied before [20] when using Cockcroft and Gault. The model fit improved with a statistically significant drop in OFV of 31 (*p* < 0.001) and a reduction of the unexplained variability in *CL* from 68 to 49%. Figure 3 illustrates the covariate effect of *eCLcr* on the typical value of *CL*, where a 10 mL/min change in *eCLcr* results in a 0.74 L/h change in *CL* for *eCLcr* values at or below 120 mL/min. The *y*-intercept, 4.2 L/h, indicates the part of cefotaxime *CL* that is not dependent on glomerular filtration represented by *eCLcr* in this dataset. Inclusion of the other covariates (e.g. burn patients) after inclusion of *eCLcr* as a covariate did not significantly improve the model.

Table 2 shows the estimated parameters and associated uncertainty for the final PK model. Parameter estimates were provided with acceptable precision, and the residual diagnostics as well as the pcVPC (Fig. 2) demonstrate the model to be an adequate description of the data. The median relative standard error (RSE) from MAP estimation was 12% for *CL* and 16% for *Vc* when using day 1 data (Online Resource 1, Table S1).

**Table 2:** Parameter estimates of the final population pharmacokinetic model, including NONMEM estimates with uncertainty, shrinkage and bootstrap median and confidence intervals (*n* = 2000)

**Parameter**	**Parameter description**	**Estimate**	(RSE%)	**Bootstrap**	
			[SHR%]	Median	95% CI
*CL* (L/h)^a^	Clearance	11.1	(8.2)	10.9	8.5–14.0
*Vc* (L)^b^	Central volume of distribution	5.13	(28)	4.84	1.5–17.6
*Vp* (L)^c^	Peripheral volume of distribution	18.2	(12)	16.8	8.0–24.8
*Q* (L/h)^d^	Inter-compartmental clearance	14.5	(19)	12.7	3.1–24.7
*θ*_cov (*eCLcr* ≤ 120)_	*eCLcr* covariate effect on *CL*	6.67	(17)	6.61	4.0–9.0
*f*_*CL*, *Vc*_	Scaling factor for IIV* CL* and *Vc*	1.31	(39)	1.30	0.2–2.5
IIV* CL* (%*CV*)	Inter-individual variability in *CL*	49	(15) [0.8]	50	36–71
IIV* Vc* (%*CV*)	Inter-individual variability in *Vc*	64^b^			
Proportional residual error (%*CV*)	Proportional residual error	33.3	(5.9) [7.8]	32.9	29–37

RSE relative standard error, SHR shrinkage on standard deviation scale, CI confidence interval, *θ* population parameter, *eCLcr* estimated creatinine clearance (mL/min), IIV inter-individual variability, *CV* coefficient of variation, *BW* body weight at intensive care unit admission, *CL*_*i*_ individual *CL*, *Vc*_*i*_ individual *Vc*, *Vp*_*i*_ individual *Vp*, *Q*_*i*_ individual *Q*, *η*1 the deviation from the typical parameter in the individual

^a^*CL*_*i*_ = (*CL* × (*BW*/92)^0.75^ × (1 + *θ*_cov (*eCLcr* ≤ 120)_ × (*eCLcr* − 94)/1000)) × *e*^*η1*^,

If *eCLcr* > 120 mL/min: *CL*_*i*_ = (*CL* × (*BW*/92)^0.75^ × (1 + *θ*_cov (*eCLcr* ≤ 120)_ × (120 − 94)/1000)) × *e*^*η*1^

^b^*Vc*_*i*_ = (*Vc* × (*BW*/92)) × *e*^*η1* × *fCL*, *Vc*^, the *CV* for *Vc* was derived as *CV* for CL times the estimated *f*_*CL*,*Vc*_

^c^*Vp*_*i*_ = *Vp* × (*BW*/92)

^d^*Q*_*i*_ = *Q* × (*BW*/92)^0.75^

## Discussion

Appropriate dosing of antibiotics in the early stage of sepsis and septic shock may be challenging due to the changing PK. Knowledge of the PK of antibiotics in the patient population is the first step towards further evaluation of target attainment and improvement of dosing recommendations. To address the lack of knowledge on cefotaxime PK in an ICU patient population, we developed a population PK model using data from cefotaxime treatment in ICU patients. Data collection started at the first day of cefotaxime treatment, thereby capturing the early treatment phase, and continued for up to three consecutive days. The study design included mid- and trough sampling of cefotaxime concentrations. Only four patients were on renal replacement therapy, and these patients were excluded from the analysis.

Our data show that cefotaxime follows a two-compartment PK model with linear elimination related to the individual *eCLcr*. These results are in line with previous descriptions of a biphasic disposition in adults [21, 22] and the findings of Urien et al. [8], where a two-compartment model with *CL* influenced by weight, plasma creatinine and age (all parts of the *eCLcr* component) best described the data in elderly patients. That study also found serum protein concentrations to be a significant covariate. However, as this is not routinely registered in clinical practice in the current ICU setting, it was not evaluated in the present study.

The resulting cefotaxime typical *CL* value (11.1 L/h for a subject with ICU weight of 92 kg and with *eCLcr* of 94 mL/min) is in line with previously reported 10–13 L/h in patients with liver disease [23] and higher than 5.4 L/h in elderly patients [8]. Clearance of 15–17 L/h in healthy adult populations [21, 22] is higher than in this ICU population as expected due to acute kidney injury in severe illness despite occasional cases of ARC.

Comparison of *V* for the central compartment (5.1 L) and the peripheral compartment (18.2 L) with healthy adults (6.5 L resp. 8.6 L) [21] and elderly patients (5.5 L resp. 5.8 L) [8] show a high peripheral volume of distribution (*Vp*) for the typical ICU patient in this study. However, higher total *V* of 26–32 L has been reported for patients with liver disease in different disease stages [23]. Koedjik et al. [24] showed a median total *V* in ICU patients on renal replacement therapy of 22.5 L, results that are in line with this current investigation. High *V* in ICU patients can be attributed to fluid leakage into the interstitial space and the use of resuscitation fluids [4], an effect that could be further pronounced by the high proportion of burn patients (35%) in this study.

The intention of this study was to create a population PK model that reflects the clinical reality and includes covariates relevant for further use in a clinical setting. The treating physicians did the dose selection, and nurses on the wards did the cefotaxime sampling, but with a controlled registration of dosing and sampling times using case report forms. Only cefotaxime, and not its metabolite, was measured in this study. This is also according to clinical practice, since the European Committee on Antimicrobial Susceptibility Testing (EUCAST) clinical breakpoint [25] only considers the parent drug in setting the breakpoint to guide clinical dosing.

The estimated *CLcr* has a modest effect on explaining the IIV in *CL* in this model. Further, at low *eCLcr*, the covariate effect of *eCLcr* on *CL* is not fully captured despite testing of different covariate and residual error models. One reason for this can be that creatinine concentrations, routinely drawn at 6 a.m. each day with occasional extra samples, were used in the modelling process. This resulted in a potential gap between the sampled cefotaxime concentrations and corresponding creatinine of up to 24 h, something that can be misleading in cases of rapid reduction of kidney function. Using a creatinine concentration from the next day and carrying the values backward could be an alternative but would then limit the use of the model for dose selection in clinic where one depends on available data before dosing. Further, even though creatinine is frequently used, it is well known that it is a poor marker in ICU patients with unstable kidney function [26]. Also, renal failure might be associated with multiple organ failure and affect non-renal components of *CL*. In an attempt to capture this, SAPS3 was tested as a potential covariate but did not improve the model further.

This analysis estimated a high unexplained variability in *Vc*. We could not find covariates to explain the variability in the final model, although day of treatment was a statistically significant covariate (*p* < 0.05) before the inclusion of *eCLcr* as a covariate on *CL*. There was no clear correlation between day of treatment and *eCLcr* (*r* = 0.17) that could help explain this. However, one reason might be lack of data for day 3 where only 34 out of the 51 patients had samples drawn due to discontinued treatment. In addition, a study period of 3 days might be too short to capture the clinical improvement and changes in *V* over time in this relatively small and heterogeneous study population.

A limitation of the study is that the study design only included mid- and trough samples, potentially resulting in that *Vc* (RSE 28%), *Vp* (RSE 12%) and/or IIV for *Vc* (IIV scaling factor RSE 39%) might not have been properly characterised. A model based on data with sampling earlier in the dosing interval could help describe *V* in more detail.

The final model results in large unexplained IIV in *CL* (49%) and *Vc* (64%). Even if IIV could potentially be reduced by the successful inclusion of more covariates, it is likely that the unexplained variability between individuals in this population will remain high and that there is a need for individualisation of cefotaxime dosing.

A limitation of this study is that external validation has not been performed. Also, the model was developed using mainly mid- and trough samples and model performance has only been evaluated for this sampling strategy. However, after evaluation of predictive performance in the local setting, this model may provide support for model-based therapeutic drug monitoring and the implementation of dose individualisation in critically ill patients.

## Conclusion

A population PK model was developed to describe cefotaxime PK and associated variability in adult ICU patients. Estimated creatinine clearance as a covariate partly explained the IIV in cefotaxime *CL*. However, the remaining unexplained IIV in *CL* and *Vc* is still high and suggests a need for dose individualisation using therapeutic drug monitoring in critically ill where the developed model, after evaluation of predictive performance, may provide support.

## Supplementary Information

Below is the link to the electronic supplementary material.

Supplementary file1 (DOCX 14 KB)

Supplementary file2 (PDF 109 KB)

## Data Availability

The datasets analysed during the current study are available from the corresponding author on reasonable request.

## Code availability

The final model code describing the final model with input, compartments, equations, initial estimates for parameters and variances as well as estimation method is included in this published article (Online Resource 2).
