# Supplementary material for: Population pharmacokinetics of cefotaxime in intensive care patients
Source: Eur J Clin Pharmacol. 2021 Oct 1;78(2):251–8. doi: 10.1007/s00228-021-03218-6 (PMC8748331; doi:10.1007/s00228-021-03218-6)
Supplement: Supplementary file 1 — Supplementary file1 (DOCX 14 KB) [file 228_2021_3218_MOESM1_ESM.docx]

**Online Resource material 1**

Population pharmacokinetics of cefotaxime in intensive care patients

European Journal of Clinical Pharmacology

Swartling M, Smekal A-K, Furebring M, Lipcsey M, Jönsson S, Nielsen EI

*Corresponding author*

Elisabet I Nielsen, Department of Pharmacy, Uppsala University. Uppsala, Sweden

elisabet.nielsen@farmaci.uu.se

**Table S1** Resulting precision (relative standard error, RSE) after maximum a posteriori (MAP) estimation of the individual PK parameters clearance (CL) and central volume of distribution (Vc) using observations from day 1 and all observations from day 1-3, respectively.

|  | CL  Observations day 1 | Vc  Observations day 1 | CL Observations  day 1-3 | Vc  Observations  day 1-3 |
| --- | --- | --- | --- | --- |
| Median RSE %  [IQR]  (range) | 12.2  [11.1-15.6] (7.3-22.6) | 15.9  [14.4-20.3]  (9.5-29.5) | 7.8  [6.6-9.1]  (6.0-19.3) | 10.2  [8.6-11.8] (7.9-25.1) |
