# Supplementary material for: Population pharmacokinetics of cefotaxime in intensive care patients
Source: Eur J Clin Pharmacol. 2021 Oct 1;78(2):251–8. doi: 10.1007/s00228-021-03218-6 (PMC8748331; doi:10.1007/s00228-021-03218-6)
Supplement: Supplementary file 2 — Supplementary file2 (PDF 109 KB) [file 228_2021_3218_MOESM2_ESM.pdf]

**Online Resource material 2**

**NONMEM code**

Population pharmacokinetics of cefotaxime in intensive care patients

European Journal of Clinical Pharmacology

Swartling M, Smekal A-K, Furebring M, Lipcsey M, Jönsson S, Nielsen EI

*Corresponding author*
Elisabet I Nielsen, Department of Pharmacy, Uppsala University. Uppsala, Sweden
elisabet.nielsen@farmaci.uu.se

```
$PROBLEM    iv intermittent inf (excl CRRT) final run

$INPUT      REF ABDAY ID DAT2=DROP TIME EVID AMT AB DV AGE KREA DIA2 DIA Sex
BWBI BWAI RATE COGBWB COGBWA BURN

$DATA      NONMEM_ACCIS5_cefmodel.csv IGNORE=@ IGNORE=(AB.EQ.1)
IGNORE=(AB.EQ.3) IGNORE=(AB.EQ.4)
IGNORE=(REF.EQ.159) IGNORE=(REF.EQ.893) IGNORE=(REF.EQ.1018)
IGNORE=(REF.EQ.1109) IGNORE=(ID.EQ.134)
IGNORE=(ID.EQ.210)IGNORE=(ID.EQ.220) IGNORE=(ID.EQ.625)

$SUBROUTINE ADVAN3 TRANS4

$PK
IF (AMT.GT.0) THEN
TDOS=TIME
TAD=0.0
ENDIF
IF (AMT.EQ.0) TAD=TIME-TDOS

  IF(COGBWA.LE.120) THEN
  CLCOV = THETA(7)*((COGBWA-94)/1000)
  ENDIF
  IF(COGBWA.GT.120) THEN
  CLCOV = THETA(7)*((120-94)/1000)
  ENDIF

  TVCL = THETA(1)*(BWAI/92)**0.75*(1+CLCOV)
  TVV1 = THETA(2)*(BWAI/92)
  TVV2 = THETA(3)*(BWAI/92)
  TVQ = THETA(4)*(BWAI/92)**0.75

  CL = TVCL*EXP(ETA(1))
  V1 = TVV1*EXP(ETA(1)*THETA(6))
  V2 = TVV2
  Q = TVQ

  S1 = V1

$THETA  (0,12)
$THETA  (0,5)
$THETA  (0,18)
$THETA  (0,14)
$THETA  0.3
$THETA  (0,1.3)
$THETA  (-6.33,7,11.77)
$OMEGA  0.2
$ERROR
  IPRED  = F
  IRES   = DV - IPRED
  W      = IPRED*THETA(5)

IF(W.EQ.0) W=1
  IWRES  = IRES/W
```

```
  Y      = IPRED + W*EPS(1)
$SIGMA  1  FIX

$ESTIMATION METHOD=1 INTER MAXEVAL=9999
$COVARIANCE PRINT=E
```
